# Supplementary material for: Optogenetic Clustering of Human IRE1 Reveals Differential Regulation of Transcription and mRNA Splice Isoform Abundance by the UPR
Source: bioRxiv. 2025 Jul 21:2025.07.16.665212. Preprint. [Version 1] doi: 10.1101/2025.07.16.665212 (PMC12330662; doi:10.1101/2025.07.16.665212)
Supplement: Supplement 1 [file media-1.pdf]

Supplemental Materials

Primer ID	Sequence
dT_BC1001_PB	AAGCAGTGGTATCAACGCAGAGTACCACATATCAGAGTGCGTTTTTTTTT TTTTTTTTTTTTTTTTTTTTTVN
dT_BC1002_PB	AAGCAGTGGTATCAACGCAGAGTACACACACAGACTGTGAGTTTTTTTT TTTTTTTTTTTTTTTTTTTTTTVN
dT_BC1003_PB	AAGCAGTGGTATCAACGCAGAGTACACACATCTCGTGAGAGTTTTTTTTT TTTTTTTTTTTTTTTTTTTTTVN
dT_BC1004_PB	AAGCAGTGGTATCAACGCAGAGTACCACGCACACACGCGCGTTTTTTTT TTTTTTTTTTTTTTTTTTTTTTVN
dT_BC1005_PB	AAGCAGTGGTATCAACGCAGAGTACCACTCGACTCTCGCGTTTTTTTTTT TTTTTTTTTTTTTTTTTTTTTVN
dT_BC1006_PB	AAGCAGTGGTATCAACGCAGAGTACCATATATATCAGCTGTTTTTTTTTTT TTTTTTTTTTTTTTTTTTTTVN
dT_BC1007_PB	AAGCAGTGGTATCAACGCAGAGTACTCTGTATCTCTATGTGTTTTTTTTTT TTTTTTTTTTTTTTTTTTTTVN
dT_BC1008_PB	AAGCAGTGGTATCAACGCAGAGTACACAGTCGAGCGCTGCGTTTTTTTT TTTTTTTTTTTTTTTTTTTTTTVN
dT_BC1009_PB	AAGCAGTGGTATCAACGCAGAGTACACACACGCGAGACAGATTTTTTTT TTTTTTTTTTTTTTTTTTTTTTVN
dT_BC1010_PB	AAGCAGTGGTATCAACGCAGAGTACACGCGCTATCTCAGAGTTTTTTTTT TTTTTTTTTTTTTTTTTTTTTVN
dT_BC1011_PB	AAGCAGTGGTATCAACGCAGAGTACCTATACGTATATCTATTTTTTTTTTTT TTTTTTTTTTTTTTTTTTTVN
dT_BC1012_PB	AAGCAGTGGTATCAACGCAGAGTACACACTAGATCGCGTGTTTTTTTTTT TTTTTTTTTTTTTTTTTTTTTVN
strand-switching primer	AAGCAGTGGTATCAACGCAGAGTAC-r(GGG)
PR2 primer	AAGCAGTGGTATCAACGCAGAGTAC
oJS023_HERPUD1_mRNA_f	TGAGCAGATTCCTCATGGTC
oJS024_HERPUD1_mRNA_r	GATCAGTGCCTTCCTGTAAGT
oJS027_SSR1_mRNA_f	TGAACCCACAGATTTGGTAGAA
oJS028_SSR1_mRNA_r	TGTTGGTAAAGCCTACCAGG
oJS031_SEC23A_mRNA_f	TTGACACTGAACATGGAGGC
oJS032_SEC23A_mRNA_r	TGCTCCAGACTCCTGCC
oJS039_TAPBP_mRNA_f	TATCTCAGTGTACACGACCCC
oJS040_TAPBP_mRNA_r	GGCTCATCTCGCAGTGTG
oJS051_KLF10_mRNA_f	GTCGAGTGTCTCCGTGC
oJS052_KLF10_mRNA_r	ATTTCCATTCTTTCCTCCGCA
oJS095_EIF4A2_exon_10_f	CAACAAGTGTCTTTGGTTAT
oJS096_EIF4A2_exon_11_r	AAATCGACCCCCTCT
oJS099_HSP90B1_exon_13_f	GAAAACTAAGGAGAGTCGT
oJS100_HSP90B1_exon_14_r	CTTTCATGATTCTCTCCAT

oJS107_HMOX1_mRNA_f1	GTGCCACCAAGTTCAAGCAG
oJS108_HMOX1_mRNA_r1	GCAACTCCTCAAAGAGCTGGA
oJS123_PLPP5_mRNA_f1	CGGCCTTCCTGGTGACG
oJS124_PLPP5_mRNA_r1	CGGCTTGGTGGGGAAATACT
oJS131_TMEM165_mRNA_f1	GGCCCGGGTCGAGAAAAT
oJS132_TMEM165_mRNA_r1	TCAGGCGGTTATAGCGCATT
oJS165_MGAT4B_mRNA_f4	CTGCTGCTCTTCTGCCTGT
oJS166_MGAT4B_mRNA_r4	CTTTCTGACACGGCCCTCTT
VB_pr166_HsXBP1_L	AGCTTTTACGAGAGAAAACTCAT
VB_pr220_HsACT_RT_L	TTCTACAATGAGCTGCGTGTG
VB_pr221_HsACT_RT_R	AGGGACATACCCCTCGTAGAT
VB_pr222_HsXBP1s_RT_R	CCTGCACCTGCTGCG
VB_pr245_HsERdj4_L	GTCGGAGGGTGCAGGATATTAG
VB_pr246_HsERdj4_R	TCAGGGTGGTACTTCATGGC
VB_pr255_HsCHOP_L	CCTCCTGGAAATGAAGAGGAAGA
VB_pr256_HsCHOP_R	TCCTGGTTCTCCCTTGGTCT
VB_pr257_HsXBP1_L	TAAGACAGCGCTTGGGGATG
VB_pr258_HsXBP1_R	TGTTCTGGAGGGGTGACAAC
VB_pr261_HsXBP1_L	ATGAGTGAGCTGGAACAGCAA
VB_pr262_HsXBP1_R	GGCCTCACTTCATTCCCCTTG

Table S1. DNA Oligo Sequences Used.

Gene ID	Category	Description
MSL2	chromatin	part of MSL complex, which regulates histone ubiquitylation. May also help respond to DNA damage.[1–3]
DLG2	Other	seems to be a scaffold protein in signal transduction complexes in certain neurons[4,5]
RBL1	chromatin	regulates chromatin modification. Related to retinoblastoma 1 (RB1), and controls cell cycle progression to some extent.[6–8]
TCF20	transcription	transcription factor that seems to be important in neural development[9,10]
FBXL20	Other	F-box protein. One of the substrate recognizing proteins in E3 ubiquitin ligases[11,12]
GATAD2B	chromatin	critical in the NURD histone deacetylation complex which represses genes.[13]
KANSL1	chromatin	unstructured scaffold protein for the nonspecific lethal NSL complex that regulates histone modification, necessary for recruitment of WDR5. necessary for proper maintenance of epigenetic cell identity. Also important for mitochondrial function [14–16]
EIF4G3	Other	part of eIF4F cap-binding complex of ribosome[17]

NPIPB12	Other	unknown function.
MED13	transcription	component of the mediator complex, which is heavily involved in RNA PolII transcription[18,19]
SLC16A4	Other	transmembrane solute transporter in the [20]monocarboxylate transporter family
LINC01297	Other	uncharacterized lncRNA
DENND5B	lipid metabolism	Rab12 GEF[21,22]
MBTD1	chromatin	Polycomb group protein. Binds to select histone modifications to regulate DNA repair pathways and transcriptional regulation [23,24]
DIP2B	Other	Possibly involved in DNA methylation, but there's not enough data to be confident.[25]
ZNF850	Other	Probably a DNA binding protein, possibly involved in CTG repeat expansion[26]
LINC-PINT	Other	tumor/proliferation suppressor[27]
TLK2	chromatin	Chromatin repair/maintenance[28]
NCOA6	transcription	coregulator of transcriptional regulators and histone modifiers[29]
LOC100190986	Other	uncharacterized transcript
CAMK1D	Other	potential regulator of Ca levels or signaling[30,31]
ASAP1	Actin	GTPase involved in active cytoskeleton remodeling, especially at motile edges/podosomes[32]
TYW1B	Other	creates wybutosine residues on tRNa.[33]
ZFHX3	transcription	repressive TF. cooperates with Smad2/3 to repress AFP, downstream of TGFbeta. Upregulated under hypoxia[34,35]
ARHGAP11B	mitochondrial function	inhibits adenine nucleotide translocase[36]
ARID1B	transcription	changes the gene targets of the chromatin remodelers SWI/SNF/BAF and inhibits Wnt/β-catenin. Important for proper cell differentiation and development. [37,38]
LINC01876	Other	uncharacterized lncRNA
LOC442028	Other	uncharacterized
CUX1	transcription	TF involved with proliferation and possibly DNA damage repair[39]
MIR34AHG	transcription	lncRNA associated with ER stress. Possibly is a precursor to miR-34a, which seems to have broad regulatory effects, including p53 repression.[40,41]
TMEM120B	lipid metabolism	encourages adipogenesis[42]
RASA4	Other	Under high intracellular Ca, Deactivates Ras, represses cancer (and other cell activities)[43,44]
DGAT2	lipid metabolism	catalyzes DAG reaction with acyl-CoAs to make trigylcerides. Seems to have overlapping role with DGAT1 for TG synthesis, but they are unrelated proteins with different affinities for substrates and cofactors, and different regulation and activities[45,46]

PVT1	transcription	seems to upregulate c-Myc and increase proliferation[47]
ITGB5	transcription	promotes metastisis and cell migration, maybe through Wnt/B-catenin pathway, smad, or TGFB[48,49]
FRMD5	Actin	Interacts with integrins[50]
AGO2	transcription	key part of the RISC silencing complex. Regulates chromatin modification through siRNA[51]
SVIL	Actin	large actin-binding protein that plays a role in motility and cytokinesis.[52,53]
ZNF782	Other	uncharacterized zinc finger protein
DOCK5	Actin	GEF of Rac1, a Rho GTPase. regulator of cytokinesis and actin dynamics.[54,55]
KRT17	Other	keratin protein
MGAT4B	glycosylation	glycan branching[56,57]
TMEM179B	mitochondrial function	ROS protection in/near mitochondia.[58]
PCBP1-AS1	transcription	seems to promote proliferation in cancers[59]
ANKDD1A	Other	uncharacterized ankyrin repeat protein. [60]
EPB41L4A-AS1	mitochondrial function	regulating glycolysis and glutaminolysis[61]
BCAM	Other	regulates integrin binding to laminin, thus altering cell adhesion[62,63]
TMEM165	ER-Golgi function	possible Ca/Mn and H+ antiporter that is needed for production of certain glycans, pulls Ca or Mn into ER[64–66]
FOXN3	transcription	seems to repress Myc and glycolysis. Seems to repress smad2-4 signaling. Seems to repress proliferation[67,68]
SIPA1L3	Actin	plays some role in actin cytoskeleton regulation[69]
ND1	mitochondrial function	critical component of mitochondia complex I, a component of the ETC. Can be downregulated in tumors to promote glycolysis over oxidative respiration[70,71]
MLLT10	transcription	activating TF that is related to development and differentiation and can directly induce colorectal cancer by promoting proliferation and invasion[72]
ASXL1	chromatin	Coordinates deubiquitylation of histones involved with polycomb silencing[73,74]
HMGA2	transcription	transcription factor that is disregulated in some cancers[75,76]
PIGQ	lipid metabolism	essential for GPI synthesis[77]
TIMP2	Other	inhibits matrix metalloproteases. Seems to be stress induced[78,79]
EEF1A2	Other	Part of the ribosome, helps with delivery of aminoacyl tRNAs to the ribosome[80]
GCLM	Other	Important for glutathione synthesis, which is one of the main ROS scavengers[81]

MRPL23	mitochondrial function	mitochondrial ribosome protein[82]
ZYX	Actin	zinc-binding phosphoprotein that functions with focal adhesions and actin cytoskeleton. Important for actin organization and cell motility[83,84]
RALB	ER-Golgi function	GTPase that seems to signal downstream of Ras. Seems to be actively involved with endomembrane autophagy and cell invasion/motility. Also improves DSB repair after irradiation[85,86]
VANGL1	Actin	scaffolding protein related to cell polarity and organization in development[87]
WAPL	chromatin	regulates cohesin binding, which is critical for both chromatin organization and chromosome segregation[88,89]
RFTN1	Other	lipid raft protein important in B cell antigen receptor function.[90,91]
LRP10	ER-Golgi function	ER-golgi localized transmembrane protein, potentially involved in trafficking of lipoproteins. Also potentially involved in lewy body diseases[92,93]
DCBLD2	Other	an orphan receptor with several proposed affiliations with cancer proliferation.[94]
SLAIN2	Actin	important for microtubule formation and organization, especially during interphase[95]
LAMP1	Other	One isoform of the most critical membrane protein in lysosomes.[96]
CAVIN1	Actin	Major component of caveolae structure[97]
TYRO3	Other	A receptor tyrosine kinase with several proposed functions[98]
AGPS	lipid metabolism	converts acyl-glycerone-3-phosphate into alkyl-glycerone-3-phosphate. Important for ether lipid synthesis[99]
FAM220A	transcription	regulates STAT3, which is a transcription factor involved in several processes.[100]
POLR3D	transcription	part of RNA PolIII[101]
CCDC6	Other	seems to be a scaffolding protein that helps with genotoxic response[102,103]
IBTK	Other	inhibits BTK kinase, which is critical in activation of B cells downstream of B-cell antigen receptor via calcium release from the ER. Also activates NFkB[104]
ACTR2	Actin	part of ARP2/3 complex, which is one of the actin nucleators that polymerize actin at the end of filaments. Partly responsible for cell motility[105]
FNDC3B	lipid metabolism	Important for adipocyte differentiation and other developmental processes. Also associated with UPR[106,107]
LTN1	Other	ubiquitylates ribosomes stalled in non-stop translation on the polyA tail[108,109]
DNAJC3	ER-Golgi function	co-chaperone for BiP[110]
HMOX1	Other	heme oxygenase that is related to ferroptosis[111,112]

OSBPL2	lipid metabolism	lipid binding protein that likely regulates lipid transport and memrane composition, and may regulate lipid droplets in conjunction with the ER[113]
PGM3	ER-Golgi function	enzyme necessary for creating UDP-GlcNAc, which is necessary for protein glycosylation[114]
ARMCX3	mitochondrial function	plays some regulatory role regarding mitochondria regulation and trafficking, especially in neuronal development[115,116]
ALG2	ER-Golgi function	mannosyltransferase important for glycosylation[117]
SRPRB	ER-Golgi function	component of the signal recognition particle receptor[118]
SLC33A1	ER-Golgi function	membrane transporter of acetyl-CoA into the ER. Necessary for ganglioside acetylation[119,120]
FKBP14	ER-Golgi function	Proline isomerase that helps folding in ER, especially for collagens[121,122]
GORASP2	ER-Golgi function	Maintains golgi structure[123,124]
HERPUD1	ER-Golgi function	ER membrane protein that is critical for ERAD[125,126]
FICD	ER-Golgi function	regulates BiP activity by AMPylating or deAMPylating it, depending on biological context.[127,128]
TRIM32	Other	multifunctional protein with several proposed rolls[129]
DNAJB9	ER-Golgi function	primarily ER chaperone that seems to either escort unfolded proteins to ERAD machinery or help them fold with BiP. Also gets localized to the nucleus in response to certain stresses.[130,131]
KLF10	transcription	a repressor of TGF-β signaling[132]
SEC24D	ER-Golgi function	One paralog of SEC24, which is a critical component of COPII vessicles.[133]
GFPT1	glycosylation	first and rate-limiting part of the hexosamine biosynthesis pathway[134,135]
TMED7	ER-Golgi function	critical for ER-golgi translocation[136]
CANX	ER-Golgi function	ER chaperone[137,138]
DNAJC10	ER-Golgi function	PDI in the ER[139]
SSR1	ER-Golgi function	signal sequence receptor, helps with translocation into the ER during translation[140]
IQCG	Actin	seems to interact with calmodulin, maybe moderating calcium levels. Or it is a microtuble nucleator/regulator. Plays a role in sperm motility[141]
TMED10	ER-Golgi function	critical for ER-golgi translocation. Seems to specifically bind GPI-anchored proteins. Co-regulated with TMED2 [136]

CYB561	mitochondrial function	membrane enzyme that does ascorbate redox, potentially involved in mitochondrial function[142]
TMED7-TICAM2	ER-Golgi function	A readthrough transcript of TMED7, which is critical for ER-golgi translocation[136,143]
SEC61A1	ER-Golgi function	part of the sec61 translocon that helps insert proteins into the ER membrane[144]
SSR3	ER-Golgi function	signal sequence receptor, helps with translocation into the ER during translation[140]
SND1	transcription	part of RISC complex. Also seems to alter alt splicing. Seems to regulate lipid metabolism genes.[145,146]
LMAN1	ER-Golgi function	membrane-bound chaperone that is critical for making certain proteins like coagulation factors F-V and -VIII. Helps transport them to the Golgi[147,148]
PDIA3	ER-Golgi function	PDI in the ER[149]
PLPP5	lipid metabolism	enzyme that is involved with phospholipid metabolism[150]
ST6GALNAC4	glycosylation	transfers sialic acid to glycan chains, preferrentially to glycoproteins[151,152]
FBXO16	Other	F-box protein. One of the substrate recognizing proteins in E3 ubiquitin ligases. May repress beta catenin and inflammation[153,154]
OSTC	glycosylation	a subunit of the oligosaccharyltransferase complex, which acts near sec61 to glycosylate peptides. Upregulated under heat stress, seems to be specific to one version of the core of the OST complex[155,156]
SERP1	ER-Golgi function	membrane protein associated with translation and translocation into the ER. Might help protect other membrane proteins from ERAD during ER stress[157,158]
CDK2AP2	chromatin	inhibits cell cycle progression, also involved with the NuRD histone modification complex[159,160]
NANS	glycosylation	catalyzes a step in the pathway for sialic acid[161]
SEC61B	ER-Golgi function	part of the sec61 translocon that helps insert proteins into the ER membrane[144]
MYDGF	Other	seems to be a proliferative signal[162]
PPIB	ER-Golgi function	Part of a complex that functions as a PDI and to hydroxylate prolyl residues in preprocollagen. Works after P4HB[163]
SEC11C	ER-Golgi function	one of the paralogs of the catalytic subunit of SPC complex, which cleaves signal sequences in the ER[164]
TMED9	ER-Golgi function	regulates COP vesicles to affect protein transportation between ER and Golgi[136]
NUCB2	Other	multi-component peptide that is cleaved into 3 parts that seem to have different functions. Nesfatin-1 seems to be the most important and is used as a hunger-related hormone[165,166]

SSR2	ER-Golgi function	signal sequence receptor, helps with translocation into the ER during translation[140]
GMPPA	glycosylation	part of the complex that produces GDP-Mannose, which is necessary for mannose glycosylation. Seems to have a regulatory role while GMPPB is catalytic[167]
P4HB	ER-Golgi function	Part of a complex that functions as a PDI and to hydroxylate prolyl residues in preprocollagen. Works before PPIB[168]
HM13	ER-Golgi function	one of the peptidases in ERAD that cleaves proteins from signal peptides during/after translocation. Has specific substrates[169]
ZSCAN31	transcription	TF. Not very well studied, but seems to get regulated in many cancers[170]
TXNDC15	ER-Golgi function	ER-resident thioredoxin related to the PDI family[171]
SMIM14	Other	uncharacterized ER membrane protein[172]

Table S2. Genes Found to be Regulated By Opto-IRE1.

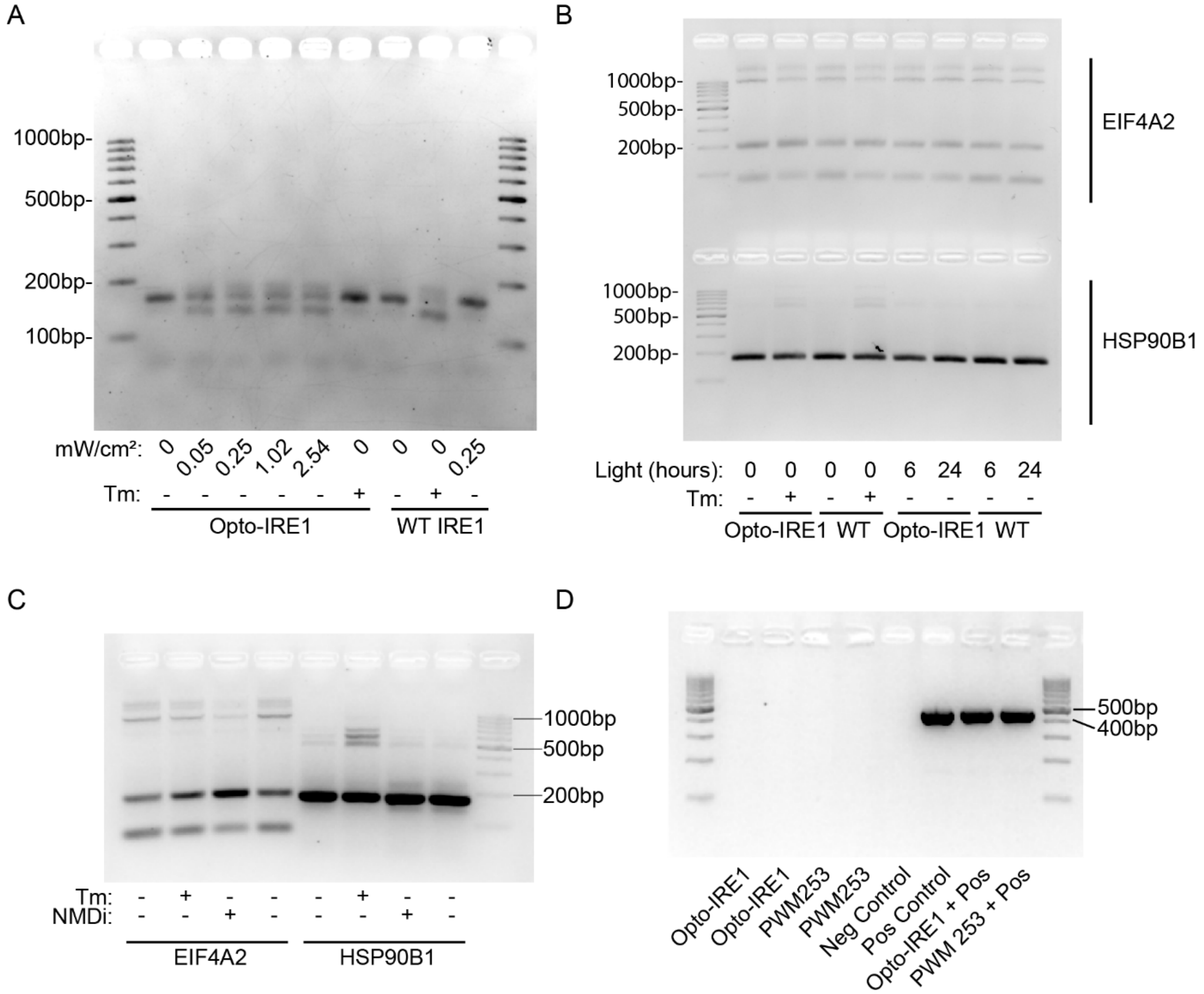

Figure S1. Uncropped Gels. (A) XBP1 splicing gel for Figure 1C. RNA was purified from Opto-IRE1 and WT IRE1 cells, reverse transcribed, and amplified with primers across the unconventional intron of XBP1 then resolved on a 3% agarose gel. (B) Gel of splicing assay for Figure 5, panels D and E. RNA was collected and reverse transcribed then amplified with primers targeting the alternatively spliced regions of the transcripts for *EIF4A2* (top half of gel) or *HSP90B1* (bottom half of gel). (C) Gel of splicing assay for Figure 6B. RNA was collected from WT cells and reverse transcribed then amplified with primers targeting the alternatively spliced regions of the transcripts for *EIF4A2* or *HSP90B1*. (D) Mycoplasma testing of Opto-IRE1 and WT cells. Cell lines were tested for mycoplasma contamination using Universal Mycoplasma Detection Kit (ATCC 301012K). The presence of a band at 434-468bp indicates the presence of mycoplasma DNA.

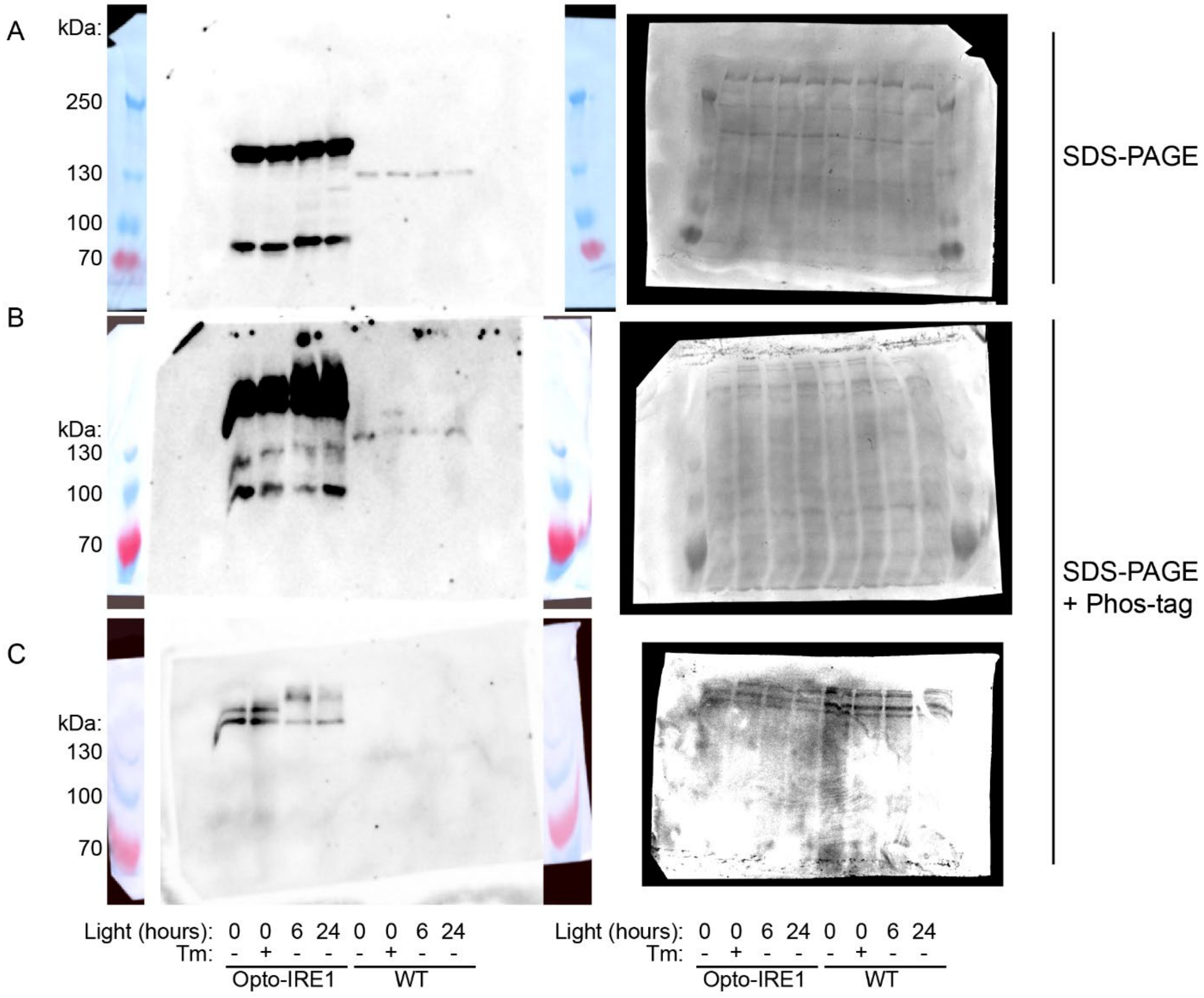

Fig S2. Uncropped Immunoblots for Figure 1B. Total protein was collected from Opto-IRE1 and WT IRE1 cells, separated by SDS-PAGE (A) or Phos-tag SDS-PAGE (B, C), and probed with anti-IRE1 antibody. 12µg of total protein was loaded per lane in panels A and B, while 10µg of total protein was loaded per lane in panel C to reduce the signal of the Opto-IRE1 bands. The WT lanes of panel B and the Opto-IRE1 lanes of panel C were used for Fig. 1B. Ponceau staining of each blot is shown on the right as a loading control.

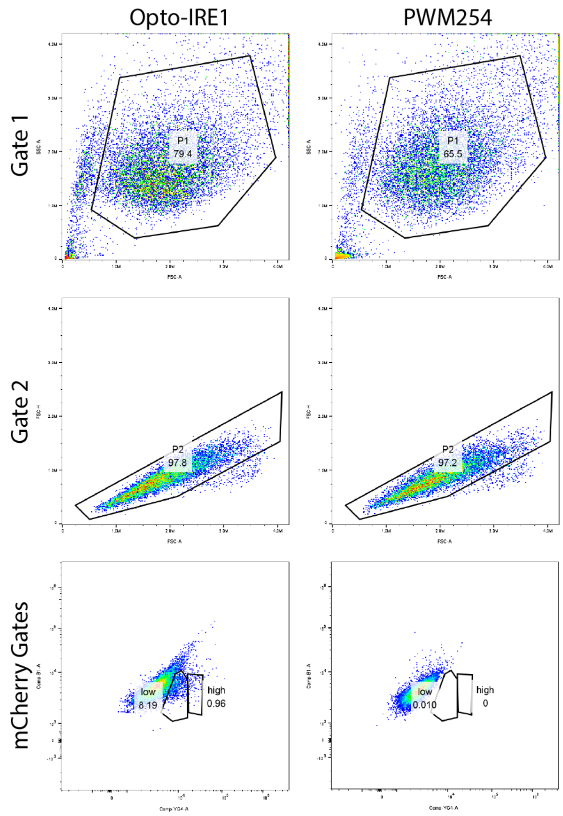

Fig S3. FACS Sorting of Opto-IRE1 and PWM254 cells. Droplets were gated twice to target single cells and then gated based on mCherry signal into low- and high-expressing populations.

A

```
Q: HSP90B1 intron-retaining sequence
S: GRCh38.p14 Chromosome 12

Q 103943266  AAAGAATTTGAGCCTCTGCTGAATTGGATGAAAGATAAAGCCCTTAAGGACAAGGTACTG  103943325
             |||||||||||||||||||||| ||||||||| |||||||||||||||||||||||||||
S 4          AAAGAATTTGAGCCTCTGCTGA-TTGGATGAA-GATAAAGCCCTTAAGGACAAGGTACTG  61

Q 103943326  TGGAAATTACAAATTGTGGAAATATTAGTATCAGCATTTAAGAGAAAGTTATTTTGTGAA  103943385
             ||||||||||||||||||||||||||||||||||||||||||||||||||||||||||||
S 62         TGGAAATTACAAATTGTGGAAATATTAGTATCAGCATTTAAGAGAAAGTTATTTTGTGAA  121

Q 103943386  CAAATTAAGCTGCAGCTGGTTACTTTGTAACCATTAGAATGGTAAAAATTTAATTAATGT  103943445
             ||||||||||||||||||||||||||||||||||||||||||||||||||||||||||||
S 122        CAAATTAAGCTGCAGCTGGTTACTTTGTAACCATTAGAATGGTAAAAATTTAATTAATGT  181

Q 103943446  AATTAAATTATTGGGAGAAAGCTTAAAACTTTCGACAATACTGCTTTGTTAATAACTTGT  103943505
             ||||||||||||||||||||||||||||||||||||||||||||||||||||||||||||
S 182        AATTAAATTATTGGGAGAAAGCTTAAAACTTTCGACAATACTGCTTTGTTAATAACTTGT  241

Q 103943506  TACAAATTAAATTTTATGTTTTTAAAAGGTGGTATTTAAACTTCTGACTAGAAAATTCAG  103943565
             ||||||||||||||||||||||||||||||||||||||||||||||||||||||||||||
S 242        TACAAATTAAATTTTATGTTTTTAAAAGGTGGTATTTAAACTTCTGACTAGAAAATTCAG  301

Q 103943566  ATTATCAAGTAAGTGCCCCTACAAATTCTCCTAAACCTTAAGAAAAGCTATTTTTATGAC  103943625
             ||||||||||||||||||||||||||||||||||||||||||||||||||||||||||||
S 302        ATTATCAAGTAAGTGCCCCTACAAATTCTCCTAAACCTTAAGAAAAGCTATTTTTATGAC  361

Q 103943626  CTGCTTCTGTGTTTATGATCTTAAGTGATAAAGTCTTAGACAGTTGAAAGACAATTGCTC  103943685
             ||||||||||||||||||||||||||||||||||||||||||||||||||||||||||||
S 362        CTGCTTCTGTGTTTATGATCTTAAGTGATAAAGTCTTAGACAGTTGAAAGACAATTGCTC  421

Q 103943686  AATGACCTTACCTGTTGATATTAATTTATATGACTTGATTTCTTTCCCTAAGATTGAAAA  103943745
             ||||||||||||||||||||||||||||||||||||||||||||||||||||||||||||
S 422        AATGACCTTACCTGTTGATATTAATTTATATGACTTGATTTCTTTCCCTAAGATTGAAAA  481

Q 103943746  GGCTGTGGTGTCTCAGCGCCTGACAGAATCTCCGTGTGCTTTGGTGGCCAGCCAGTACGG  103943805
             ||||||||||||||||||||||||||||||||||||||||||||||||||||||||||||
S 482        GGCTGTGGTGTCTCAGCGCCTGACAGAATCTCCGTGTGCTTTGGTGGCCAGCCAGTACGG  541

Q 103943806  ATGGTCTGGCAACATGGAGAGAATCATGAAA  103943836
             ||||||||||||||||||||| |||||| ||
S 542        ATGGTCTGGCAACATGGAGAG-ATCATGGAA  571
```

B

```
Q: EIF4A2 intron-retaining sequence
S: GRCh38.p14 Chomosome 3

Q 8          ACCCATCGTGAA--CTATATTCACAG  31
             ||| ||||||||  ||||||||||||
S 186787857  ACCAATCGTGAAAACTATATTCACAG  1867878829

Q 32         GAGTCGATAGCAGCAGTTGGTGACGAGATGGCACTCAGAAACGGCGTTGACGTAAT  87
             ||||||||||||||||||||||||||||||||||||||||||||||||||||||||
S  186788310 GAGTCGATAGCAGCAGTTGGTGACGAGATGGCACTCAGAAACGGCGTTGACGTAAT  186788365

Q 88         TTAGGACGTGGAATCATAAGCGAAACAGCACACTGTTTGAATAAAGAGCGAGTCG  142
             |||||||||||||||||||||||||||||||||||||||||||||||||||||||
S 186788366  TTAGGACGTGGAATCATAAGCGAAACAGCACACTGTTTGAATAAAGAGCGAGTCG  186788420
```

Fig S4. Sequencing Results of the Intron-retaining PCR Products for *EIF4A2* and *HSP90B1*. For EIF4A2, the ~200bp band was purified, sequenced, and aligned to the human genome. For *HSP90B1*, the ~600bp band was purified, sequenced, and aligned to the human genome using BLAST[173,174].

References

(1) Cai, Y.; Jin, J.; Swanson, S. K.; Cole, M. D.; Choi, S. H.; Florens, L.; Washburn, M. P.; Conaway, J. W.; Conaway, R. C. Subunit Composition and Substrate Specificity of a MOF-Containing Histone Acetyltransferase Distinct from the Male-Specific Lethal (MSL) Complex *. *J. Biol. Chem.* **2010**, *285* (7), 4268–4272. https://doi.org/10.1074/jbc.C109.087981.

(2) Lai, Z.; Moravcová, S.; Canitrot, Y.; Andrzejewski, L. P.; Walshe, D. M.; Rea, S. Msl2 Is a Novel Component of the Vertebrate DNA Damage Response. *PLOS ONE* **2013**, *8* (7), e68549. https://doi.org/10.1371/journal.pone.0068549.

(3) Marsh, D. J.; Dickson, K.-A. Writing Histone Monoubiquitination in Human Malignancy—The Role of RING Finger E3 Ubiquitin Ligases. *Genes* **2019**, *10* (1), 67. https://doi.org/10.3390/genes10010067.

(4) Zhu, J.; Shang, Y.; Zhang, M. Mechanistic Basis of MAGUK-Organized Complexes in Synaptic Development and Signalling. *Nat. Rev. Neurosci.* **2016**, *17* (4), 209–223. https://doi.org/10.1038/nrn.2016.18.

(5) Roberts, S.; Delury, C.; Marsh, E. The PDZ Protein Discs-Large (DLG): The 'Jekyll and Hyde' of the Epithelial Polarity Proteins. *FEBS J.* **2012**, *279* (19), 3549–3558. https://doi.org/10.1111/j.1742-4658.2012.08729.x.

(6) Lee, M. H.; Williams, B. O.; Mulligan, G.; Mukai, S.; Bronson, R. T.; Dyson, N.; Harlow, E.; Jacks, T. Targeted Disruption of P107: Functional Overlap between P107 and Rb. *Genes Dev.* **1996**, *10* (13), 1621–1632. https://doi.org/10.1101/gad.10.13.1621.

(7) Modi, S.; Kubo, A.; Oie, H.; Coxon, A. B.; Rehmatulla, A.; Kaye, F. J. Protein Expression of the RB-Related Gene Family and SV40 Large T Antigen in Mesothelioma and Lung Cancer. *Oncogene* **2000**, *19* (40), 4632–4639. https://doi.org/10.1038/sj.onc.1203815.

(8) Henley, S. A.; Dick, F. A. The Retinoblastoma Family of Proteins and Their Regulatory Functions in the Mammalian Cell Division Cycle. *Cell Div.* **2012**, *7* (1), 10. https://doi.org/10.1186/1747-1028-7-10.

(9) Feng, C.; Zhao, J.; Ji, F.; Su, L.; Chen, Y.; Jiao, J. TCF20 Dysfunction Leads to Cortical Neurogenesis Defects and Autistic-like Behaviors in Mice. *EMBO Rep.* **2020**, *21* (8), e49239. https://doi.org/10.15252/embr.201949239.

(10) Vetrini, F.; McKee, S.; Rosenfeld, J. A.; Suri, M.; Lewis, A. M.; Nugent, K. M.; Roeder, E.; Littlejohn, R. O.; Holder, S.; Zhu, W.; Alaimo, J. T.; Graham, B.; Harris, J. M.; Gibson, J. B.; Pastore, M.; McBride, K. L.; Komara, M.; Al-Gazali, L.; Al Shamsi, A.; Fanning, E. A.; Wierenga, K. J.; Scott, D. A.; Ben-Neriah, Z.; Meiner, V.; Cassuto, H.; Elpeleg, O.; Holder, J. L.; Burrage, L. C.; Seaver, L. H.; Van Maldergem, L.; Mahida, S.; Soul, J. S.; Marlatt, M.; Matyakhina, L.; Vogt, J.; Gold, J.-A.; Park, S.-M.; Varghese, V.; Lampe, A. K.; Kumar, A.; Lees, M.; Holder-Espinasse, M.; McConnell, V.; Bernhard, B.; Blair, E.; Harrison, V.; Muzny, D. M.; Gibbs, R. A.; Elsea, S. H.; Posey, J. E.; Bi, W.; Lalani, S.; Xia, F.; Yang, Y.; Eng, C. M.; Lupski, J. R.; Liu, P.; The DDD study. De Novo and Inherited TCF20 Pathogenic Variants Are Associated with Intellectual Disability, Dysmorphic Features, Hypotonia, and Neurological Impairments with Similarities to Smith–Magenis Syndrome. *Genome Med.* **2019**, *11* (1), 12. https://doi.org/10.1186/s13073-019-0623-0.

(11) Wang, W.; Li, Y.; Wang, L.; Chen, X.; Lan, T.; Wang, C.; Chen, S.; Yu, S. FBXL20 Promotes Synaptic Impairment in Depression Disorder *via* Degrading Vesicle-Associated Proteins. *J. Affect. Disord.* **2024**, *349*, 132–144. https://doi.org/10.1016/j.jad.2024.01.055.

(12) Mason, B.; Laman, H. The FBXL Family of F-Box Proteins: Variations on a Theme. *Open Biol.* **2020**, *10* (11), 200319. https://doi.org/10.1098/rsob.200319.

(13) Lin, Y.; Yu, L.; Xu, Q.; Qiu, P.; Zhang, Y.; Dong, X.; Yan, G.; Sun, H.; Cao, G. GATAD2B Is Required for Pre-Implantation Embryonic Development by Regulating Zygotic Genome Activation. *Cell Prolif.* **2024**, *57* (9), e13647. https://doi.org/10.1111/cpr.13647.

(14) Dias, J.; Nguyen, N. V.; Georgiev, P.; Gaub, A.; Brettschneider, J.; Cusack, S.; Kadlec, J.; Akhtar, A. Structural Analysis of the KANSL1/WDR5/KANSL2 Complex Reveals That WDR5 Is Required for Efficient Assembly and Chromatin Targeting of the NSL Complex. *Genes Dev.* **2014**, *28* (9), 929–942. https://doi.org/10.1101/gad.240200.114.

(15) Tsang, T. H.; Wiese, M.; Helmstädter, M.; Stehle, T.; Seyfferth, J.; Shvedunova, M.; Holz, H.; Walz, G.; Akhtar, A. Transcriptional Regulation by the NSL Complex Enables Diversification of IFT Functions in Ciliated versus Nonciliated Cells. *Sci. Adv.* **2023**, *9* (34), eadh5598. https://doi.org/10.1126/sciadv.adh5598.

(16) Chatterjee, A.; Seyfferth, J.; Lucci, J.; Gilsbach, R.; Preissl, S.; Böttinger, L.; Mårtensson, C. U.; Panhale, A.; Stehle, T.; Kretz, O.; Sahyoun, A. H.; Avilov, S.; Eimer, S.; Hein, L.; Pfanner, N.; Becker, T.; Akhtar, A. MOF Acetyl Transferase Regulates Transcription and Respiration in Mitochondria. *Cell* **2016**, *167* (3), 722-738.e23. https://doi.org/10.1016/j.cell.2016.09.052.

(17) Shestakova, E. D.; Smirnova, V. V.; Shatsky, I. N.; Terenin, I. M. Specific Mechanisms of Translation Initiation in Higher Eukaryotes: The eIF4G2 Story. *RNA* **2023**, *29* (3), 282–299. https://doi.org/10.1261/rna.079462.122.

(18) Schiano, C.; Napoli, C. Mediator Complex: Update of Key Insights into Transcriptional Regulation of Ancestral Framework and Its Role in Cardiovascular Diseases. *Eur. J. Med. Res.* **2025**, *30* (1), 507. https://doi.org/10.1186/s40001-025-02720-2.

(19) Harper, T. M.; Taatjes, D. J. The Complex Structure and Function of Mediator. *J. Biol. Chem.* **2018**, *293* (36), 13778–13785. https://doi.org/10.1074/jbc.R117.794438.

(20) Halestrap, A. P. The SLC16 Gene Family – Structure, Role and Regulation in Health and Disease. *Mol. Aspects Med.* **2013**, *34* (2), 337–349. https://doi.org/10.1016/j.mam.2012.05.003.

(21) Gordon, S. M.; Neufeld, E. B.; Yang, Z.; Pryor, M.; Freeman, L. A.; Fan, X.; Kullo, I. J.; Biesecker, L. G.; Remaley, A. T. DENND5B Regulates Intestinal Triglyceride Absorption and Body Mass. *Sci. Rep.* **2019**, *9* (1), 3597. https://doi.org/10.1038/s41598-019-40296-0.

(22) Mobilia, M.; Whitus, C.; Karakashian, A.; Lu, H. S.; Daugherty, A.; Gordon, S. M. Dennd5b-Deficient Mice Are Resistant to PCSK9-Induced Hypercholesterolemia and Diet-Induced Hepatic Steatosis. *J. Lipid Res.* **2022**, *63* (12), 100296. https://doi.org/10.1016/j.jlr.2022.100296.

(23) Zhang, H.; Devoucoux, M.; Song, X.; Li, L.; Ayaz, G.; Cheng, H.; Tempel, W.; Dong, C.; Loppnau, P.; Côté, J.; Min, J. Structural Basis for EPC1-Mediated Recruitment of MBTD1 into the NuA4/TIP60 Acetyltransferase Complex. *Cell Rep.* **2020**, *30* (12), 3996-4002.e4. https://doi.org/10.1016/j.celrep.2020.03.003.

(24) Takubo, K.; Htun, P. W.; Ueda, T.; Sera, Y.; Iwasaki, M.; Koizumi, M.; Shiroshita, K.; Kobayashi, H.; Haraguchi, M.; Watanuki, S.; Honda, Z.; Yamasaki, N.; Nakamura-Ishizu, A.; Arai, F.; Motoyama, N.; Hatta, T.; Natsume, T.; Suda, T.; Honda, H. MBTD1 Preserves Adult Hematopoietic Stem Cell Pool Size and Function. *Proc. Natl. Acad. Sci.* **2023**, *120* (32), e2206860120. https://doi.org/10.1073/pnas.2206860120.

(25) Larsson, C.; Ali, M. A.; Pandzic, T.; Lindroth, A. M.; He, L.; Sjöblom, T. Loss of DIP2C in RKO Cells Stimulates Changes in DNA Methylation and Epithelial-Mesenchymal Transition. *BMC Cancer* **2017**, *17* (1), 487. https://doi.org/10.1186/s12885-017-3472-5.

(26) Kamon, M.; Wakatsuki, S.; Nakamori, M.; Takahashi, M. P.; Mori-Yoshimura, M.; Komaki, H.; Araki, T. Identification of ZNF850 as a Novel CTG Repeat Expansion-Related Gene in Myotonic Dystrophy Type 1 Patient-Derived iPSCs. *Hum. Mol. Genet.* **2025**, *34* (4), 327–337. https://doi.org/10.1093/hmg/ddae186.

(27) Bukhari, I.; Khan, M. R.; Hussain, M. A.; Thorne, R. F.; Yu, Y.; Zhang, B.; Zheng, P.; Mi, Y. PINTology: A Short History of the lncRNA LINC-PINT in Different Diseases. *WIREs RNA* **2022**, *13* (4), e1705. https://doi.org/10.1002/wrna.1705.
(28) Mortuza, G. B.; Hermida, D.; Pedersen, A.-K.; Segura-Bayona, S.; López-Méndez, B.; Redondo, P.; Rüther, P.; Pozdnyakova, I.; Garrote, A. M.; Muñoz, I. G.; Villamor-Payà, M.; Jauset, C.; Olsen, J. V.; Stracker, T. H.; Montoya, G. Molecular Basis of Tousled-Like Kinase 2 Activation. *Nat. Commun.* **2018**, *9* (1), 2535. https://doi.org/10.1038/s41467-018-04941-y.
(29) Mahajan, M. A.; Samuels, H. H. Nuclear Receptor Coactivator/Coregulator NCoA6(NRC) Is a Pleiotropic Coregulator Involved in Transcription, Cell Survival, Growth and Development. *Nucl. Recept. Signal.* **2008**, *6* (1), nrs.06002. https://doi.org/10.1621/nrs.06002.
(30) Verploegen, S.; Lammers, J.-W. J.; Koenderman, L.; Coffer, P. J. Identification and Characterization of CKLiK, a Novel Granulocyte Ca++/Calmodulin-Dependent Kinase. *Blood* **2000**, *96* (9), 3215–3223. https://doi.org/10.1182/blood.V96.9.3215.
(31) Wang, L.; Huang, J.; Jiang, M.; Chen, Q.; Jiang, Z.; Feng, H. CAMK1 Phosphoinositide Signal-Mediated Protein Sorting and Transport Network in Human Hepatocellular Carcinoma (HCC) by Biocomputation. *Cell Biochem. Biophys.* **2014**, *70* (2), 1011–1016. https://doi.org/10.1007/s12013-014-0011-8.
(32) Randazzo, P. A.; Andrade, J.; Miura, K.; Brown, M. T.; Long, Y.-Q.; Stauffer, S.; Roller, P.; Cooper, J. A. The Arf GTPase-Activating Protein ASAP1 Regulates the Actin Cytoskeleton. *Proc. Natl. Acad. Sci.* **2000**, *97* (8), 4011–4016. https://doi.org/10.1073/pnas.070552297.
(33) Perche-Letuvée, P.; Molle, T.; Forouhar, F.; Mulliez, E.; Atta, M. Wybutosine Biosynthesis: Structural and Mechanistic Overview. *RNA Biol.* **2014**, *11* (12), 1508–1518. https://doi.org/10.4161/15476286.2014.992271.
(34) Sakata, N.; Kaneko, S.; Ikeno, S.; Miura, Y.; Nakabayashi, H.; Dong, X.-Y.; Dong, J.-T.; Tamaoki, T.; Nakano, N.; Itoh, S. TGF-β Signaling Cooperates with AT Motif-Binding Factor-1 for Repression of the α-Fetoprotein Promoter. *J. Signal Transduct.* **2014**, *2014*, 970346. https://doi.org/10.1155/2014/970346.
(35) Fu, C.; An, N.; Liu, J.; A., J.; Zhang, B.; Liu, M.; Zhang, Z.; Fu, L.; Tian, X.; Wang, D.; Dong, J.-T. The Transcription Factor ZFHX3 Is Crucial for the Angiogenic Function of Hypoxia-Inducible Factor 1α in Liver Cancer Cells. *J. Biol. Chem.* **2020**, *295* (20), 7060–7074. https://doi.org/10.1074/jbc.RA119.012131.
(36) Namba, T.; Dóczi, J.; Pinson, A.; Xing, L.; Kalebic, N.; Wilsch-Bräuninger, M.; Long, K. R.; Vaid, S.; Lauer, J.; Bogdanova, A.; Borgonovo, B.; Shevchenko, A.; Keller, P.; Drechsel, D.; Kurzchalia, T.; Wimberger, P.; Chinopoulos, C.; Huttner, W. B. Human-Specific ARHGAP11B Acts in Mitochondria to Expand Neocortical Progenitors by Glutaminolysis. *Neuron* **2020**, *105* (5), 867-881.e9. https://doi.org/10.1016/j.neuron.2019.11.027.
(37) Vasileiou, G.; Ekici, A. B.; Uebe, S.; Zweier, C.; Hoyer, J.; Engels, H.; Behrens, J.; Reis, A.; Hadjihannas, M. V. Chromatin-Remodeling-Factor ARID1B Represses Wnt/β-Catenin Signaling. *Am. J. Hum. Genet.* **2015**, *97* (3), 445–456. https://doi.org/10.1016/j.ajhg.2015.08.002.
(38) Moffat, J. J.; Jung, E.-M.; Ka, M.; Smith, A. L.; Jeon, B. T.; Santen, G. W. E.; Kim, W.-Y. The Role of ARID1B, a BAF Chromatin Remodeling Complex Subunit, in Neural Development and Behavior. *Prog. Neuropsychopharmacol. Biol. Psychiatry* **2019**, *89*, 30–38. https://doi.org/10.1016/j.pnpbp.2018.08.021.
(39) Liu, N.; Sun, Q.; Wan, L.; Wang, X.; Feng, Y.; Luo, J.; Wu, H. CUX1, A Controversial Player in Tumor Development. *Front. Oncol.* **2020**, *10*. https://doi.org/10.3389/fonc.2020.00738.
(40) Chen, J.; Shen, L.; Yang, Y. Endoplasmic Reticulum Stress Related IncRNA Signature Predicts the Prognosis and Immune Response Evaluation of Uterine Corpus Endometrial Carcinoma. *Front. Oncol.* **2023**, *12*. https://doi.org/10.3389/fonc.2022.1064223.

(41) Navarro, F.; Lieberman, J. miR-34 and P53: New Insights into a Complex Functional Relationship. *PLOS ONE* **2015**, *10* (7), e0132767. https://doi.org/10.1371/journal.pone.0132767.
(42) Batrakou, D. G.; Heras, J. I. de las; Czapiewski, R.; Mouras, R.; Schirmer, E. C. TMEM120A and B: Nuclear Envelope Transmembrane Proteins Important for Adipocyte Differentiation. *PLOS ONE* **2015**, *10* (5), e0127712. https://doi.org/10.1371/journal.pone.0127712.
(43) Dai, Y.; Walker, S. A.; de Vet, E.; Cook, S.; Welch, H. C. E.; Lockyer, P. J. Ca2+-Dependent Monomer and Dimer Formation Switches CAPRI Protein between Ras GTPase-Activating Protein (GAP) and RapGAP Activities. *J. Biol. Chem.* **2011**, *286* (22), 19905–19916. https://doi.org/10.1074/jbc.M110.201301.
(44) Lockyer, P. J.; Kupzig, S.; Cullen, P. J. CAPRI Regulates Ca2+-Dependent Inactivation of the Ras-MAPK Pathway. *Curr. Biol.* **2001**, *11* (12), 981–986. https://doi.org/10.1016/S0960-9822(01)00261-5.
(45) Zammit, V. A. Hepatic Triacylglycerol Synthesis and Secretion: DGAT2 as the Link between Glycaemia and Triglyceridaemia. *Biochem. J.* **2013**, *451* (1), 1–12. https://doi.org/10.1042/BJ20121689.
(46) Chitraju, C.; Walther, T. C.; Farese, R. V. The Triglyceride Synthesis Enzymes DGAT1 and DGAT2 Have Distinct and Overlapping Functions in Adipocytes. *J. Lipid Res.* **2019**, *60* (6), 1112–1120. https://doi.org/10.1194/jlr.M093112.
(47) Onagoruwa, O. T.; Pal, G.; Ochu, C.; Ogunwobi, O. O. Oncogenic Role of PVT1 and Therapeutic Implications. *Front. Oncol.* **2020**, *10*. https://doi.org/10.3389/fonc.2020.00017.
(48) Chen, Z.; Fang, Y.; Zhong, S.; Lin, S.; Yang, X.; Chen, S. ITGB5 Is a Prognostic Factor in Colorectal Cancer and Promotes Cancer Progression and Metastasis through the Wnt Signaling Pathway. *Sci. Rep.* **2025**, *15* (1), 9225. https://doi.org/10.1038/s41598-025-93081-7.
(49) Shi, W.; He, J.; Huang, Y.; Zeng, Z.; Feng, Z.; Xu, H.; Nie, Y. Integrin B5 Enhances the Malignancy of Human Colorectal Cancer by Increasing the TGF-β Signaling. *Anticancer. Drugs* **2021**, *32* (7), 717. https://doi.org/10.1097/CAD.0000000000001050.
(50) Hu, J.; Niu, M.; Li, X.; Lu, D.; Cui, J.; Xu, W.; Li, G.; Zhan, J.; Zhang, H. FERM Domain-Containing Protein FRMD5 Regulates Cell Motility via Binding to Integrin B5 Subunit and ROCK1. *FEBS Lett.* **2014**, *588* (23), 4348–4356. https://doi.org/10.1016/j.febslet.2014.10.012.
(51) Li, X.; Wang ,Xueying; Cheng ,Zeneng; and Zhu, Q. AGO2 and Its Partners: A Silencing Complex, a Chromatin Modulator, and New Features. *Crit. Rev. Biochem. Mol. Biol.* **2020**, *55* (1), 33–53. https://doi.org/10.1080/10409238.2020.1738331.
(52) Javary, J.; Goupil, E.; Soulez, M.; Kanshin, E.; Bouchard, A.; Seternes, O.-M.; Thibault, P.; Labbé, J.-C.; Meloche, S. Phosphoproteomic Analysis Identifies Supervillin as an ERK3 Substrate Regulating Cytokinesis and Cell Ploidy. *J. Cell. Physiol.* **2024**, *239* (3), e30938. https://doi.org/10.1002/jcp.30938.
(53) Son, K.; Smith, T. C.; Luna, E. J. Supervillin Binds the Rac/Rho-GEF Trio and Increases Trio-Mediated Rac1 Activation. *Cytoskeleton* **2015**, *72* (1), 47–64. https://doi.org/10.1002/cm.21210.
(54) Ogawa, K.; Tanaka, Y.; Uruno, T.; Duan, X.; Harada, Y.; Sanematsu, F.; Yamamura, K.; Terasawa, M.; Nishikimi, A.; Côté, J.-F.; Fukui, Y. DOCK5 Functions as a Key Signaling Adaptor That Links FcεRI Signals to Microtubule Dynamics during Mast Cell Degranulation. *J. Exp. Med.* **2014**, *211* (7), 1407–1419. https://doi.org/10.1084/jem.20131926.
(55) Guimbal, S.; Morel, A.; Guérit, D.; Chardon, M.; Blangy, A.; Vives, V. Dock5 Is a New Regulator of Microtubule Dynamic Instability in Osteoclasts. *Biol. Cell* **2019**, *111* (11), 271–283. https://doi.org/10.1111/boc.201900014.
(56) Oguri, S.; Yoshida, A.; Minowa, M. T.; Takeuchi, M. Kinetic Properties and Substrate Specificities of Two Recombinant Human N-Acetylglucosaminyltransferase-IV Isozymes. *Glycoconj. J.* **2006**, *23* (7), 473–480. https://doi.org/10.1007/s10719-006-6216-3.

(57) Takamatsu, S.; Antonopoulos, A.; Ohtsubo, K.; Ditto, D.; Chiba, Y.; Le, D. T.; Morris, H. R.; Haslam, S. M.; Dell, A.; Marth, J. D.; Taniguchi, N. Physiological and Glycomic Characterization of N-Acetylglucosaminyltransferase-IVa and -IVb Double Deficient Mice. *Glycobiology* **2010**, *20* (4), 485–497. https://doi.org/10.1093/glycob/cwp200.
(58) He, Z.; Zhang, Y.; Zhang, H.; Zhou, C.; Ma, Q.; Deng, P.; Lu, M.; Mou, Z.; Lin, M.; Yang, L.; Li, Y.; Yue, Y.; Pi, H.; Lu, Y.; He, M.; Zhang, L.; Chen, C.; Zhou, Z.; Yu, Z. NAC Antagonizes Arsenic-Induced Neurotoxicity through TMEM179 by Inhibiting Oxidative Stress in Oli-Neu Cells. *Ecotoxicol. Environ. Saf.* **2021**, *223*, 112554. https://doi.org/10.1016/j.ecoenv.2021.112554.
(59) Zhang, B.; Zhang, M.; Shen, C.; Liu, G.; Zhang, F.; Hou, J.; Yao, W. LncRNA PCBP1-AS1-Mediated AR/AR-V7 Deubiquitination Enhances Prostate Cancer Enzalutamide Resistance. *Cell Death Dis.* **2021**, *12* (10), 856. https://doi.org/10.1038/s41419-021-04144-2.
(60) Zhao, P.; Fu, Y.; Yang, X.; Abdoulaye, H. A.; Rauniyar, R.; Peng, J.; Wang, M.; Wang, H.; Ning, M.; Chen, Y.; Huang, Y. ANKDD1A May Serve as a Critical Gene in the Immune Microenvironment of Breast Cancer. *Transl. Cancer Res.* **2021**, *10* (3), 1358–1367. https://doi.org/10.21037/tcr-20-2685.
(61) Liao, M.; Liao, W.; Xu, N.; Li, B.; Liu, F.; Zhang, S.; Wang, Y.; Wang, S.; Zhu, Y.; Chen, D.; Xie, W.; Jiang, Y.; Cao, L.; Yang, B. B.; Zhang, Y. LncRNA EPB41L4A-AS1 Regulates Glycolysis and Glutaminolysis by Mediating Nucleolar Translocation of HDAC2. *eBioMedicine* **2019**, *41*, 200–213. https://doi.org/10.1016/j.ebiom.2019.01.035.
(62) Kikkawa, Y.; Ogawa, T.; Sudo, R.; Yamada, Y.; Katagiri, F.; Hozumi, K.; Nomizu, M.; Miner, J. H. The Lutheran/Basal Cell Adhesion Molecule Promotes Tumor Cell Migration by Modulating Integrin-Mediated Cell Attachment to Laminin-511 Protein*. *J. Biol. Chem.* **2013**, *288* (43), 30990–31001. https://doi.org/10.1074/jbc.M113.486456.
(63) Sivakumar, S.; Lieber, S.; Librizzi, D.; Keber, C.; Sommerfeld, L.; Finkernagel, F.; Roth, K.; Reinartz, S.; Bartsch, J. W.; Graumann, J.; Müller-Brüsselbach, S.; Müller, R. Basal Cell Adhesion Molecule Promotes Metastasis-Associated Processes in Ovarian Cancer. *Clin. Transl. Med.* **2023**, *13* (1), e1176. https://doi.org/10.1002/ctm2.1176.
(64) Khan, S.; Sbeity, M.; Foulquier, F.; Barré, L.; Ouzzine, M. TMEM165 a New Player in Proteoglycan Synthesis: Loss of TMEM165 Impairs Elongation of Chondroitin- and Heparan-Sulfate Glycosaminoglycan Chains of Proteoglycans and Triggers Early Chondrocyte Differentiation and Hypertrophy. *Cell Death Dis.* **2021**, *13* (1), 11. https://doi.org/10.1038/s41419-021-04458-1.
(65) Lebredonchel, E.; Houdou, M.; Potelle, S.; de Bettignies, G.; Schulz, C.; Krzewinski Recchi, M.-A.; Lupashin, V.; Legrand, D.; Klein, A.; Foulquier, F. Dissection of TMEM165 Function in Golgi Glycosylation and Its Mn2+ Sensitivity. *Biochimie* **2019**, *165*, 123–130. https://doi.org/10.1016/j.biochi.2019.07.016.
(66) Demaegd, D.; Foulquier, F.; Colinet, A.-S.; Gremillon, L.; Legrand, D.; Mariot, P.; Peiter, E.; Van Schaftingen, E.; Matthijs, G.; Morsomme, P. Newly Characterized Golgi-Localized Family of Proteins Is Involved in Calcium and pH Homeostasis in Yeast and Human Cells. *Proc. Natl. Acad. Sci.* **2013**, *110* (17), 6859–6864. https://doi.org/10.1073/pnas.1219871110.
(67) Karanth, S.; Zinkhan, E. K.; Hill, J. T.; Yost, H. J.; Schlegel, A. FOXN3 Regulates Hepatic Glucose Utilization. *Cell Rep.* **2016**, *15* (12), 2745–2755. https://doi.org/10.1016/j.celrep.2016.05.056.
(68) Sun, J.; Li, H.; Huo, Q.; Cui, M.; Ge, C.; Zhao, F.; Tian, H.; Chen, T.; Yao, M.; Li, J. The Transcription Factor FOXN3 Inhibits Cell Proliferation by Downregulating E2F5 Expression in Hepatocellular Carcinoma Cells. *Oncotarget* **2016**, *7* (28), 43534–43545. https://doi.org/10.18632/oncotarget.9780.
(69) Greenlees, R.; Mihelec, M.; Yousoof, S.; Speidel, D.; Wu, S. K.; Rinkwitz, S.; Prokudin, I.; Perveen, R.; Cheng, A.; Ma, A.; Nash, B.; Gillespie, R.; Loebel, D. A. F.; Clayton-Smith, J.; Lloyd, I. C.; Grigg, J. R.; Tam, P. P. L.; Yap, A. S.; Becker, T. S.; Black, G. C. M.; Semina, E.; Jamieson, R. V. Mutations in

SIPA1L3 Cause Eye Defects through Disruption of Cell Polarity and Cytoskeleton Organization. *Hum. Mol. Genet.* **2015**, *24* (20), 5789–5804. https://doi.org/10.1093/hmg/ddv298.
(70) Schuler, F.; Casida, J. E. Functional Coupling of PSST and ND1 Subunits in NADH:Ubiquinone Oxidoreductase Established by Photoaffinity Labeling. *Biochim. Biophys. Acta BBA - Bioenerg.* **2001**, *1506* (1), 79–87. https://doi.org/10.1016/S0005-2728(01)00183-9.
(71) Lim, S. C.; Hroudová, J.; Van Bergen, N. J.; Sanchez, M. I. G. L.; Trounce, I. A.; McKenzie, M. Loss of Mitochondrial DNA-Encoded Protein ND1 Results in Disruption of Complex I Biogenesis during Early Stages of Assembly. *FASEB J.* **2016**, *30* (6), 2236–2248. https://doi.org/10.1096/fj.201500137R.
(72) Jing, X.; Wu, H.; Cheng, X.; Chen, X.; Zhang, Y.; Shi, M.; Zhang, T.; Wang, X.; Zhao, R. MLLT10 Promotes Tumor Migration, Invasion, and Metastasis in Human Colorectal Cancer. *Scand. J. Gastroenterol.* **2018**, *53* (8), 964–971. https://doi.org/10.1080/00365521.2018.1481521.
(73) Cho, Y.-S.; Kim, E.-J.; Park, U.-H.; Sin, H.-S.; Um, S.-J. Additional Sex Comb-like 1 (ASXL1), in Cooperation with SRC-1, Acts as a Ligand-Dependent Coactivator for Retinoic Acid Receptor *. *J. Biol. Chem.* **2006**, *281* (26), 17588–17598. https://doi.org/10.1074/jbc.M512616200.
(74) Campagne, A.; Lee, M.-K.; Zielinski, D.; Michaud, A.; Le Corre, S.; Dingli, F.; Chen, H.; Shahidian, L. Z.; Vassilev, I.; Servant, N.; Loew, D.; Pasmant, E.; Postel-Vinay, S.; Wassef, M.; Margueron, R. BAP1 Complex Promotes Transcription by Opposing PRC1-Mediated H2A Ubiquitylation. *Nat. Commun.* **2019**, *10*, 348. https://doi.org/10.1038/s41467-018-08255-x.
(75) Zhang, S.; Mo, Q.; Wang, X. Oncological Role of HMGA2 (Review). *Int. J. Oncol.* **2019**, *55* (4), 775–788. https://doi.org/10.3892/ijo.2019.4856.
(76) Cattaruzzi, G.; Altamura, S.; Tessari, M. A.; Rustighi, A.; Giancotti, V.; Pucillo, C.; Manfioletti, G. The Second AT-Hook of the Architectural Transcription Factor HMGA2 Is Determinant for Nuclear Localization and Function. *Nucleic Acids Res.* **2007**, *35* (6), 1751–1760. https://doi.org/10.1093/nar/gkl1106.
(77) Tiede, A.; Daniels, R. J.; Higgs, D. R.; Mehrein, Y.; Schmidt, R. E.; Schubert, J. The Human *GPI1* Gene Is Required for Efficient Glycosylphosphatidylinositol Biosynthesis. *Gene* **2001**, *271* (2), 247–254. https://doi.org/10.1016/S0378-1119(01)00510-8.
(78) Peeney, D.; Liu, Y.; Lazaroff, C.; Gurung, S.; Stetler-Stevenson, W. G. Unravelling the Distinct Biological Functions and Potential Therapeutic Applications of TIMP2 in Cancer. *Carcinogenesis* **2022**, *43* (5), 405–418. https://doi.org/10.1093/carcin/bgac037.
(79) Wang, Z.; Soloway, P. D. TIMP-1 and TIMP-2 Perform Different Functions in Vivo. *Ann. N. Y. Acad. Sci.* **1999**, *878* (1), 519–521. https://doi.org/10.1111/j.1749-6632.1999.tb07714.x.
(80) Anand, N.; Murthy, S.; Amann, G.; Wernick, M.; Porter, L. A.; Cukier, I. H.; Collins, C.; Gray, J. W.; Diebold, J.; Demetrick, D. J.; Lee, J. M. Protein Elongation Factor EEF1A2 Is a Putative Oncogene in Ovarian Cancer. *Nat. Genet.* **2002**, *31* (3), 301–305. https://doi.org/10.1038/ng904.
(81) Franklin, C. C.; Backos, D. S.; Mohar, I.; White, C. C.; Forman, H. J.; Kavanagh, T. J. Structure, Function, and Post-Translational Regulation of the Catalytic and Modifier Subunits of Glutamate Cysteine Ligase. *Mol. Aspects Med.* **2009**, *30* (1), 86–98. https://doi.org/10.1016/j.mam.2008.08.009.
(82) Koc, E. C.; Burkhart, W.; Blackburn, K.; Moyer, M. B.; Schlatzer, D. M.; Moseley, A.; Spremulli, L. L. The Large Subunit of the Mammalian Mitochondrial Ribosome: ANALYSIS OF THE COMPLEMENT OF RIBOSOMAL PROTEINS PRESENT *. *J. Biol. Chem.* **2001**, *276* (47), 43958–43969. https://doi.org/10.1074/jbc.M106510200.
(83) Drees, B. E.; Andrews, K. M.; Beckerle, M. C. Molecular Dissection of Zyxin Function Reveals Its Involvement in Cell Motility. *J. Cell Biol.* **1999**, *147* (7), 1549–1560. https://doi.org/10.1083/jcb.147.7.1549.

(84) Kotb, A.; Hyndman, M. E.; Patel, T. R. The Role of Zyxin in Regulation of Malignancies. *Heliyon* **2018**, *4* (7). https://doi.org/10.1016/j.heliyon.2018.e00695.

(85) Kidd, A. R.; Snider, J. L.; Martin, T. D.; Graboski, S. F.; Der, C. J.; Cox, A. D. Ras-Related Small GTPases RalA and RalB Regulate Cellular Survival After Ionizing Radiation. *Int. J. Radiat. Oncol.* **2010**, *78* (1), 205–212. https://doi.org/10.1016/j.ijrobp.2010.03.023.

(86) Singh, M. K.; Martin, A. P. J.; Joffre, C.; Zago, G.; Camonis, J.; Coppey, M.; Parrini, M. C. Localization of RalB Signaling at Endomembrane Compartments and Its Modulation by Autophagy. *Sci. Rep.* **2019**, *9* (1), 8910. https://doi.org/10.1038/s41598-019-45443-1.

(87) Hatakeyama, J.; Wald, J. H.; Printsev, I.; Ho, H.-Y. H.; Carraway, K. L. Vangl1 and Vangl2: Planar Cell Polarity Components with a Developing Role in Cancer. **2014**. https://doi.org/10.1530/ERC-14-0141.

(88) Liu, N. Q.; Maresca, M.; van den Brand, T.; Braccioli, L.; Schijns, M. M. G. A.; Teunissen, H.; Bruneau, B. G.; Nora, E. P.; de Wit, E. WAPL Maintains a Cohesin Loading Cycle to Preserve Cell-Type-Specific Distal Gene Regulation. *Nat. Genet.* **2021**, *53* (1), 100–109. https://doi.org/10.1038/s41588-020-00744-4.

(89) Tedeschi, A.; Wutz, G.; Huet, S.; Jaritz, M.; Wuensche, A.; Schirghuber, E.; Davidson, I. F.; Tang, W.; Cisneros, D. A.; Bhaskara, V.; Nishiyama, T.; Vaziri, A.; Wutz, A.; Ellenberg, J.; Peters, J.-M. Wapl Is an Essential Regulator of Chromatin Structure and Chromosome Segregation. *Nature* **2013**, *501* (7468), 564–568. https://doi.org/10.1038/nature12471.

(90) Saeki, K.; Miura, Y.; Aki, D.; Kurosaki, T.; Yoshimura, A. The B Cell-specific Major Raft Protein, Raftlin, Is Necessary for the Integrity of Lipid Raft and BCR Signal Transduction. *EMBO J.* **2003**, *22* (12), 3015–3026. https://doi.org/10.1093/emboj/cdg293.

(91) Tatematsu, M.; Yoshida, R.; Morioka, Y.; Ishii, N.; Funami, K.; Watanabe, A.; Saeki, K.; Seya, T.; Matsumoto, M. Raftlin Controls Lipopolysaccharide-Induced TLR4 Internalization and TICAM-1 Signaling in a Cell Type–Specific Manner. *J. Immunol.* **2016**, *196* (9), 3865–3876. https://doi.org/10.4049/jimmunol.1501734.

(92) Boucher, R.; Larkin, H.; Brodeur, J.; Gagnon, H.; Thériault, C.; Lavoie, C. Intracellular Trafficking of LRP9 Is Dependent on Two Acidic Cluster/Dileucine Motifs. *Histochem. Cell Biol.* **2008**, *130* (2), 315–327. https://doi.org/10.1007/s00418-008-0436-5.

(93) Brodeur, J.; Larkin, H.; Boucher, R.; Thériault, C.; St-Louis, S. C.; Gagnon, H.; Lavoie, C. Calnuc Binds to LRP9 and Affects Its Endosomal Sorting. *Traffic* **2009**, *10* (8), 1098–1114. https://doi.org/10.1111/j.1600-0854.2009.00933.x.

(94) Nie, L.; Guo, X.; Esmailzadeh, L.; Zhang, J.; Asadi, A.; Collinge, M.; Li, X.; Kim, J.-D.; Woolls, M.; Jin, S.-W.; Dubrac, A.; Eichmann, A.; Simons, M.; Bender, J. R.; Sadeghi, M. M. Transmembrane Protein ESDN Promotes Endothelial VEGF Signaling and Regulates Angiogenesis. *J. Clin. Invest.* **2013**, *123* (12), 5082–5097. https://doi.org/10.1172/JCI67752.

(95) van der Vaart, B.; Manatschal, C.; Grigoriev, I.; Olieric, V.; Gouveia, S. M.; Bjelić, S.; Demmers, J.; Vorobjev, I.; Hoogenraad, C. C.; Steinmetz, M. O.; Akhmanova, A. SLAIN2 Links Microtubule plus End–Tracking Proteins and Controls Microtubule Growth in Interphase. *J. Cell Biol.* **2011**, *193* (6), 1083–1099. https://doi.org/10.1083/jcb.201012179.

(96) Eskelinen, E.-L. Roles of LAMP-1 and LAMP-2 in Lysosome Biogenesis and Autophagy. *Mol. Aspects Med.* **2006**, *27* (5), 495–502. https://doi.org/10.1016/j.mam.2006.08.005.

(97) Kovtun, O.; Tillu, V. A.; Ariotti, N.; Parton, R. G.; Collins, B. M. Cavin Family Proteins and the Assembly of Caveolae. *J. Cell Sci.* **2015**, *128* (7), 1269–1278. https://doi.org/10.1242/jcs.167866.

(98) Smart, S. K.; Vasileiadi, E.; Wang, X.; DeRyckere, D.; Graham, D. K. The Emerging Role of TYRO3 as a Therapeutic Target in Cancer. *Cancers* **2018**, *10* (12), 474. https://doi.org/10.3390/cancers10120474.

(99) Benjamin, D. I.; Cozzo, A.; Ji, X.; Roberts, L. S.; Louie, S. M.; Mulvihill, M. M.; Luo, K.; Nomura, D. K. Ether Lipid Generating Enzyme AGPS Alters the Balance of Structural and Signaling Lipids to Fuel Cancer Pathogenicity. *Proc. Natl. Acad. Sci.* **2013**, *110* (37), 14912–14917. https://doi.org/10.1073/pnas.1310894110.
(100) Ren, F.; Su, F.; Ning, H.; Wang, Y.; Geng, Y.; Feng, Y.; Wang, Y.; Zhang, Y.; Jin, Z.; Li, Y.; Jia, B.; Chang, Z. SIPAR Negatively Regulates STAT3 Signaling and Inhibits Progression of Melanoma. *Cell. Signal.* **2013**, *25* (11), 2272–2280. https://doi.org/10.1016/j.cellsig.2013.07.023.
(101) Ramsay, E. P.; Abascal-Palacios, G.; Daiß, J. L.; King, H.; Gouge, J.; Pilsl, M.; Beuron, F.; Morris, E.; Gunkel, P.; Engel, C.; Vannini, A. Structure of Human RNA Polymerase III. *Nat. Commun.* **2020**, *11* (1), 6409. https://doi.org/10.1038/s41467-020-20262-5.
(102) Cerrato, A.; Merolla, F.; Morra, F.; Celetti, A. CCDC6: The Identity of a Protein Known to Be Partner in Fusion. *Int. J. Cancer* **2018**, *142* (7), 1300–1308. https://doi.org/10.1002/ijc.31106.
(103) Merolla, F.; Luise, C.; Muller, M. T.; Pacelli, R.; Fusco, A.; Celetti, A. Loss of CCDC6, the First Identified RET Partner Gene, Affects pH2AX S139 Levels and Accelerates Mitotic Entry upon DNA Damage. *PLOS ONE* **2012**, *7* (5), e36177. https://doi.org/10.1371/journal.pone.0036177.
(104) Liu, W.; Quinto, I.; Chen, X.; Palmieri, C.; Rabin, R. L.; Schwartz, O. M.; Nelson, D. L.; Scala, G. Direct Inhibition of Bruton's Tyrosine Kinase by IBtk, a Btk-Binding Protein. *Nat. Immunol.* **2001**, *2* (10), 939–946. https://doi.org/10.1038/ni1001-939.
(105) Welch, M. D.; Iwamatsu, A.; Mitchison, T. J. Actin Polymerization Is Induced by Arp2/3 Protein Complex at the Surface of Listeria Monocytogenes. *Nature* **1997**, *385* (6613), 265–269. https://doi.org/10.1038/385265a0.
(106) Tominaga, K.; Kondo, C.; Johmura, Y.; Nishizuka, M.; Imagawa, M. The Novel Gene *Fad*104, Containing a Fibronectin Type III Domain, Has a Significant Role in Adipogenesis. *FEBS Lett.* **2004**, *577* (1), 49–54. https://doi.org/10.1016/j.febslet.2004.09.062.
(107) Han, B.; Wang, H.; Zhang, J.; Tian, J. FNDC3B Is Associated with ER Stress and Poor Prognosis in Cervical Cancer. *Oncol. Lett.* **2020**, *19* (1), 406–414. https://doi.org/10.3892/ol.2019.11098.
(108) Bengtson, M. H.; Joazeiro, C. A. P. Role of a Ribosome-Associated E3 Ubiquitin Ligase in Protein Quality Control. *Nature* **2010**, *467* (7314), 470–473. https://doi.org/10.1038/nature09371.
(109) Crowder, J. J.; Geigges, M.; Gibson, R. T.; Fults, E. S.; Buchanan, B. W.; Sachs, N.; Schink, A.; Kreft, S. G.; Rubenstein, E. M. Rkr1/Ltn1 Ubiquitin Ligase-Mediated Degradation of Translationally Stalled Endoplasmic Reticulum Proteins *. *J. Biol. Chem.* **2015**, *290* (30), 18454–18466. https://doi.org/10.1074/jbc.M115.663559.
(110) Rutkowski, D. T.; Kang, S.-W.; Goodman, A. G.; Garrison, J. L.; Taunton, J.; Katze, M. G.; Kaufman, R. J.; Hegde, R. S. The Role of p58IPK in Protecting the Stressed Endoplasmic Reticulum. *Mol. Biol. Cell* **2007**, *18* (9), 3681–3691. https://doi.org/10.1091/mbc.e07-03-0272.
(111) Meng, Z.; Liang, H.; Zhao, J.; Gao, J.; Liu, C.; Ma, X.; Liu, J.; Liang, B.; Jiao, X.; Cao, J.; Wang, Y. HMOX1 Upregulation Promotes Ferroptosis in Diabetic Atherosclerosis. *Life Sci.* **2021**, *284*, 119935. https://doi.org/10.1016/j.lfs.2021.119935.
(112) Bekeschus, S.; Freund, E.; Wende, K.; Gandhirajan, R. K.; Schmidt, A. Hmox1 Upregulation Is a Mutual Marker in Human Tumor Cells Exposed to Physical Plasma-Derived Oxidants. *Antioxidants* **2018**, *7* (11), 151. https://doi.org/10.3390/antiox7110151.
(113) Olkkonen, V. M.; Koponen, A.; Arora, A. OSBP-Related Protein 2 (ORP2): Unraveling Its Functions in Cellular Lipid/Carbohydrate Metabolism, Signaling and F-Actin Regulation. *J. Steroid Biochem. Mol. Biol.* **2019**, *192*, 105298. https://doi.org/10.1016/j.jsbmb.2019.01.016.
(114) Greig, K. T.; Antonchuk, J.; Metcalf, D.; Morgan, P. O.; Krebs, D. L.; Zhang, J.-G.; Hacking, D. F.; Bode, L.; Robb, L.; Kranz, C.; de Graaf, C.; Bahlo, M.; Nicola, N. A.; Nutt, S. L.; Freeze, H. H.; Alexander, W. S.; Hilton, D. J.; Kile, B. T. Agm1/Pgm3-Mediated Sugar Nucleotide Synthesis Is

Essential for Hematopoiesis and Development. *Mol. Cell. Biol.* **2007**, *27* (16), 5849–5859. https://doi.org/10.1128/MCB.00802-07.
(115) López-Doménech, G.; Serrat, R.; Mirra, S.; D'Aniello, S.; Somorjai, I.; Abad, A.; Vitureira, N.; García-Arumí, E.; Alonso, M. T.; Rodriguez-Prados, M.; Burgaya, F.; Andreu, A. L.; García-Sancho, J.; Trullas, R.; Garcia-Fernàndez, J.; Soriano, E. The Eutherian Armcx Genes Regulate Mitochondrial Trafficking in Neurons and Interact with Miro and Trak2. *Nat. Commun.* **2012**, *3* (1), 814. https://doi.org/10.1038/ncomms1829.
(116) Izquierdo-Villalba, I.; Mirra, S.; Manso, Y.; Parcerisas, A.; Rubio, J.; Del Valle, J.; Gil-Bea, F. J.; Ulloa, F.; Herrero-Lorenzo, M.; Verdaguer, E.; Benincá, C.; Castro-Torres, R. D.; Rebollo, E.; Marfany, G.; Auladell, C.; Navarro, X.; Enríquez, J. A.; López de Munain, A.; Soriano, E.; Aragay, A. M. A Mammalian-Specific Alex3/Gαq Protein Complex Regulates Mitochondrial Trafficking, Dendritic Complexity, and Neuronal Survival. *Sci. Signal.* **2024**, *17* (822), eabq1007. https://doi.org/10.1126/scisignal.abq1007.
(117) O'Reilly, M. K.; Zhang, G.; Imperiali, B. In Vitro Evidence for the Dual Function of Alg2 and Alg11: Essential Mannosyltransferases in N-Linked Glycoprotein Biosynthesis. *Biochemistry* **2006**, *45* (31), 9593–9603. https://doi.org/10.1021/bi060878o.
(118) Schwartz, T.; Blobel, G. Structural Basis for the Function of the β Subunit of the Eukaryotic Signal Recognition Particle Receptor. *Cell* **2003**, *112* (6), 793–803. https://doi.org/10.1016/S0092-8674(03)00161-2.
(119) Kanamori, A.; Nakayama, J.; Fukuda, M. N.; Stallcup, W. B.; Sasaki, K.; Fukuda, M.; Hirabayashi, Y. Expression Cloning and Characterization of a cDNA Encoding a Novel Membrane Protein Required for the Formation of O-Acetylated Ganglioside: A Putative Acetyl-CoA Transporter. *Proc. Natl. Acad. Sci. U. S. A.* **1997**, *94* (7), 2897–2902. https://doi.org/10.1073/pnas.94.7.2897.
(120) Jonas, M. C.; Pehar, M.; Puglielli, L. AT-1 Is the ER Membrane Acetyl-CoA Transporter and Is Essential for Cell Viability. *J. Cell Sci.* **2010**, *123* (Pt 19), 3378–3388. https://doi.org/10.1242/jcs.068841.
(121) Ishikawa, Y.; Bächinger, H. P. A Substrate Preference for the Rough Endoplasmic Reticulum Resident Protein FKBP22 during Collagen Biosynthesis. *J. Biol. Chem.* **2014**, *289* (26), 18189–18201. https://doi.org/10.1074/jbc.M114.561944.
(122) Boudko, S. P.; Ishikawa, Y.; Nix, J.; Chapman, M. S.; Bächinger, H. P. Structure of Human Peptidyl-Prolyl Cis–Trans Isomerase FKBP22 Containing Two EF-Hand Motifs. *Protein Sci.* **2014**, *23* (1), 67–75. https://doi.org/10.1002/pro.2391.
(123) Zhang, Y.; Seemann, J. Rapid Degradation of GRASP55 and GRASP65 Reveals Their Immediate Impact on the Golgi Structure. *J. Cell Biol.* **2020**, *220* (1), e202007052. https://doi.org/10.1083/jcb.202007052.
(124) Grond, R.; Veenendaal, T.; Duran, J. M.; Raote, I.; van Es, J. H.; Corstjens, S.; Delfgou, L.; El Haddouti, B.; Malhotra, V.; Rabouille, C. The Function of GORASPs in Golgi Apparatus Organization in Vivo. *J. Cell Biol.* **2020**, *219* (9), e202004191. https://doi.org/10.1083/jcb.202004191.
(125) Huang, C.-H.; Chu, Y.-R.; Ye, Y.; Chen, X. Role of HERP and a HERP-Related Protein in HRD1-Dependent Protein Degradation at the Endoplasmic Reticulum*. *J. Biol. Chem.* **2014**, *289* (7), 4444–4454. https://doi.org/10.1074/jbc.M113.519561.
(126) Okuda-Shimizu, Y.; Hendershot, L. M. Characterization of an ERAD Pathway for Nonglycosylated BiP Substrates, Which Require Herp. *Mol. Cell* **2007**, *28* (4), 544–554. https://doi.org/10.1016/j.molcel.2007.09.012.
(127) Casey, A. K.; Stewart, N. M.; Zaidi, N.; Gray, H. F.; Cox, A.; Fields, H. A.; Orth, K. FicD Regulates Adaptation to the Unfolded Protein Response in the Murine Liver. *Biochimie* **2024**, *225*, 114–124. https://doi.org/10.1016/j.biochi.2024.05.012.

(128) Preissler, S.; Rato, C.; Perera, L. A.; Saudek, V.; Ron, D. FICD Acts Bifunctionally to AMPylate and De-AMPylate the Endoplasmic Reticulum Chaperone BiP. *Nat. Struct. Mol. Biol.* **2017**, *24* (1), 23–29. https://doi.org/10.1038/nsmb.3337.
(129) Bawa, S.; Piccirillo, R.; Geisbrecht, E. R. TRIM32: A Multifunctional Protein Involved in Muscle Homeostasis, Glucose Metabolism, and Tumorigenesis. *Biomolecules* **2021**, *11* (3), 408. https://doi.org/10.3390/biom11030408.
(130) Daverkausen-Fischer, L.; Pröls, F. The Function of the Co-Chaperone ERdj4 in Diverse (Patho-)Physiological Conditions. *Cell. Mol. Life Sci.* **2021**, *79* (1), 9. https://doi.org/10.1007/s00018-021-04082-4.
(131) Lai, C. W.; Otero, J. H.; Hendershot, L. M.; Snapp, E. ERdj4 Protein Is a Soluble Endoplasmic Reticulum (ER) DnaJ Family Protein That Interacts with ER-Associated Degradation Machinery *. *J. Biol. Chem.* **2012**, *287* (11), 7969–7978. https://doi.org/10.1074/jbc.M111.311290.
(132) Memon, A.; Lee, W. K. KLF10 as a Tumor Suppressor Gene and Its TGF-β Signaling. *Cancers* **2018**, *10* (6), 161. https://doi.org/10.3390/cancers10060161.
(133) Chatterjee, S.; Choi, A. J.; Frankel, G. A Systematic Review of Sec24 Cargo Interactome. *Traffic* **2021**, *22* (12), 412–424. https://doi.org/10.1111/tra.12817.
(134) Marshall, S.; Bacote, V.; Traxinger, R. R. Discovery of a Metabolic Pathway Mediating Glucose-Induced Desensitization of the Glucose Transport System. Role of Hexosamine Biosynthesis in the Induction of Insulin Resistance. *J. Biol. Chem.* **1991**, *266* (8), 4706–4712.
(135) Ruegenberg, S.; Horn, M.; Pichlo, C.; Allmeroth, K.; Baumann, U.; Denzel, M. S. Loss of GFAT-1 Feedback Regulation Activates the Hexosamine Pathway That Modulates Protein Homeostasis. *Nat. Commun.* **2020**, *11* (1), 687. https://doi.org/10.1038/s41467-020-14524-5.
(136) Zhou, L.; Li, H.; Yao, H.; Dai, X.; Gao, P.; Cheng, H. TMED Family Genes and Their Roles in Human Diseases. *Int. J. Med. Sci.* **2023**, *20* (13), 1732–1743. https://doi.org/10.7150/ijms.87272.
(137) Bergeron, J. J. M.; Brenner, M. B.; Thomas, D. Y.; Williams, D. B. Calnexin: A Membrane-Bound Chaperone of the Endoplasmic Reticulum. *Trends Biochem. Sci.* **1994**, *19* (3), 124–128. https://doi.org/10.1016/0968-0004(94)90205-4.
(138) Paskevicius, T.; Farraj, R. A.; Michalak, M.; Agellon, L. B. Calnexin, More Than Just a Molecular Chaperone. *Cells* **2023**, *12* (3), 403. https://doi.org/10.3390/cells12030403.
(139) Robinson, P. J.; Pringle, M. A.; Fleming, B.; Bulleid, N. J. Distinct Role of ERp57 and ERdj5 as a Disulfide Isomerase and Reductase during ER Protein Folding. *J. Cell Sci.* **2023**, *136* (2), jcs260656. https://doi.org/10.1242/jcs.260656.
(140) Russo, A. Understanding the Mammalian TRAP Complex Function(s). *Open Biol.* **2020**, *10* (5), 190244. https://doi.org/10.1098/rsob.190244.
(141) Li, R.-K.; Tan, J.-L.; Chen, L.-T.; Feng, J.-S.; Liang, W.-X.; Guo, X.-J.; Liu, P.; Chen, Z.; Sha, J.-H.; Wang, Y.-F.; Chen, S.-J. Iqcg Is Essential for Sperm Flagellum Formation in Mice. *PLOS ONE* **2014**, *9* (5), e98053. https://doi.org/10.1371/journal.pone.0098053.
(142) Asard, H.; Barbaro, R.; Trost, P.; Bérczi, A. Cytochromes B561: Ascorbate-Mediated Trans-Membrane Electron Transport. *Antioxid. Redox Signal.* **2013**, *19* (9), 1026–1035. https://doi.org/10.1089/ars.2012.5065.
(143) Doyle, S. L.; Husebye, H.; Connolly, D. J.; Espevik, T.; O'Neill, L. A. J.; McGettrick, A. F. The GOLD Domain-Containing Protein TMED7 Inhibits TLR4 Signalling from the Endosome upon LPS Stimulation. *Nat. Commun.* **2012**, *3* (1), 707. https://doi.org/10.1038/ncomms1706.
(144) Lang, S.; Pfeffer, S.; Lee, P.-H.; Cavalié, A.; Helms, V.; Förster, F.; Zimmermann, R. An Update on Sec61 Channel Functions, Mechanisms, and Related Diseases. *Front. Physiol.* **2017**, *8*. https://doi.org/10.3389/fphys.2017.00887.
(145) Cappellari, M.; Bielli, P.; Paronetto, M. P.; Ciccosanti, F.; Fimia, G. M.; Saarikettu, J.; Silvennoinen, O.; Sette, C. The Transcriptional Co-Activator SND1 Is a Novel Regulator of Alternative Splicing in

Prostate Cancer Cells. *Oncogene* **2014**, *33* (29), 3794–3802. https://doi.org/10.1038/onc.2013.360.
(146) Navarro-Imaz, H.; Ochoa, B.; García-Arcos, I.; Martínez, M. J.; Chico, Y.; Fresnedo, O.; Rueda, Y. Molecular and Cellular Insights into the Role of SND1 in Lipid Metabolism. *Biochim. Biophys. Acta BBA - Mol. Cell Biol. Lipids* **2020**, *1865* (5), 158589. https://doi.org/10.1016/j.bbalip.2019.158589.
(147) Cunningham, M. A.; Pipe, S. W.; Zhang, B.; Hauri, H.-P.; Ginsburg, D.; Kaufman, R. J. LMAN1 Is a Molecular Chaperone for the Secretion of Coagulation Factor VIII1. *J. Thromb. Haemost.* **2003**, *1* (11), 2360–2367. https://doi.org/10.1046/j.1538-7836.2003.00415.x.
(148) Zheng, C.; Liu, H.; Yuan, S.; Zhou, J.; Zhang, B. Molecular Basis of LMAN1 in Coordinating LMAN1-MCFD2 Cargo Receptor Formation and ER-to-Golgi Transport of FV/FVIII. *Blood* **2010**, *116* (25), 5698–5706. https://doi.org/10.1182/blood-2010-04-278325.
(149) Mahmood, F.; Xu, R.; Awan, M. U. N.; Song, Y.; Han, Q.; Xia, X.; Zhang, J. PDIA3: Structure, Functions and Its Potential Role in Viral Infections. *Biomed. Pharmacother.* **2021**, *143*, 112110. https://doi.org/10.1016/j.biopha.2021.112110.
(150) Takeuchi, M.; Harigai, M.; Momohara, S.; Ball, E.; Abe, J.; Furuichi, K.; Kamatani, N. Cloning and Characterization of DPPL1 and DPPL2, Representatives of a Novel Type of Mammalian Phosphatidate Phosphatase. *Gene* **2007**, *399* (2), 174–180. https://doi.org/10.1016/j.gene.2007.05.009.
(151) Man, D.; Jiang, Y.; Zhang, D.; Wu, J.; Ding, B.; Liu, H.; Xu, G.; Lu, J.; Ru, J.; Tong, R.; Zheng, S.; Chen, D.; Wu, J. ST6GALNAC4 Promotes Hepatocellular Carcinogenesis by Inducing Abnormal Glycosylation. *J. Transl. Med.* **2023**, *21* (1), 420. https://doi.org/10.1186/s12967-023-04191-7.
(152) Isaji, T.; Gu, J. Novel Regulatory Mechanisms of N-Glycan Sialylation: Implication of Integrin and Focal Adhesion Kinase in the Regulation. *Biochim. Biophys. Acta BBA - Gen. Subj.* **2024**, *1868* (6), 130617. https://doi.org/10.1016/j.bbagen.2024.130617.
(153) Paul, D.; Islam, S.; Manne, R. K.; Dinesh, U.; Malonia, S. K.; Maity, B.; Boppana, R.; Rapole, S.; Shetty, P. K.; Santra, M. K. F-Box Protein FBXO16 Functions as a Tumor Suppressor by Attenuating Nuclear β-Catenin Function. *J. Pathol.* **2019**, *248* (3), 266–279. https://doi.org/10.1002/path.5252.
(154) Sugimoto-Ishige, A.; Jodo, A.; Tanaka, T. Fbxo16 Mediates Degradation of NF-κB P65 Subunit and Inhibits Inflammatory Response in Dendritic Cells. *Front. Immunol.* **2025**, *16*. https://doi.org/10.3389/fimmu.2025.1524110.
(155) Chen, C.-M.; Tseng, C.-N.; Cho, J. J.; Lee, Y.-Z.; Kao, C.-L.; Cheng, Y.-B.; Hong, Y.-R.; Cho, C.-L. Heat Shock Induces Expression of OSTC/DC2, a Novel Subunit of Oligosaccharyltransferase, *in Vitro* and *in Vivo*. *Kaohsiung J. Med. Sci.* **2014**, *30* (5), 219–223. https://doi.org/10.1016/j.kjms.2014.01.003.
(156) Shrimal, S.; Cherepanova, N. A.; Gilmore, R. DC2 and KCP2 Mediate the Interaction between the Oligosaccharyltransferase and the ER Translocon. *J. Cell Biol.* **2017**, *216* (11), 3625–3638. https://doi.org/10.1083/jcb.201702159.
(157) Jung, S.; Hyun, J.; Nah, J.; Han, J.; Kim, S.-H.; Park, J.; Oh, Y.; Gwon, Y.; Moon, S.; Jo, D.-G.; Jung, Y.-K. SERP1 Is an Assembly Regulator of γ-Secretase in Metabolic Stress Conditions. *Sci. Signal.* **2020**, *13* (623), eaax8949. https://doi.org/10.1126/scisignal.aax8949.
(158) Yamaguchi, A.; Hori, O.; Stern, D. M.; Hartmann, E.; Ogawa, S.; Tohyama, M. Stress-Associated Endoplasmic Reticulum Protein 1 (Serp1)/Ribosome-Associated Membrane Protein 4 (Ramp4) Stabilizes Membrane Proteins during Stress and Facilitates Subsequent Glycosylation. *J. Cell Biol.* **1999**, *147* (6), 1195–1204. https://doi.org/10.1083/jcb.147.6.1195.
(159) Spruijt, C. G.; Gräwe, C.; Kleinendorst, S. C.; Baltissen, M. P. A.; Vermeulen, M. Cross-Linking Mass Spectrometry Reveals the Structural Topology of Peripheral NuRD Subunits Relative to the Core Complex. *FEBS J.* **2021**, *288* (10), 3231–3245. https://doi.org/10.1111/febs.15650.

(160) Liu, Q.; Liu, X.; Gao, J.; Shi, X.; Hu, X.; Wang, S.; Luo, Y. Overexpression of DOC-1R Inhibits Cell Cycle G1/S Transition by Repressing CDK2 Expression and Activation. *Int. J. Biol. Sci.* **2013**, *9* (6), 541–549. https://doi.org/10.7150/ijbs.5763.

(161) Willems, A. P.; Sun, L.; Schulz, M. A.; Tian, W.; Ashikov, A.; van Scherpenzeel, M.; Hermans, E.; Clausen, H.; Yang, Z.; Lefeber, D. J. Activity of *N*-Acylneuraminate-9-Phosphatase (NANP) Is Not Essential for *de Novo* Sialic Acid Biosynthesis. *Biochim. Biophys. Acta BBA - Gen. Subj.* **2019**, *1863* (10), 1471–1479. https://doi.org/10.1016/j.bbagen.2019.05.011.

(162) Wang, Y.; Zhang, Y.; Li, J.; Zhao, R.; Long, X.; Li, C.; Liu, W.; Chen, W.; Shi, B. Role of Mydgf in the Regulation of Hypoxia/Reoxygenation-Induced Apoptosis in Cardiac Microvascular Endothelial Cells. *Vitro Cell. Dev. Biol. - Anim.* **2022**, *58* (8), 669–678. https://doi.org/10.1007/s11626-022-00709-3.

(163) Wu, J.; Zhang, W.; Xia, L.; Feng, L.; Shu, Z.; Zhang, J.; Ye, W.; Zeng, N.; Zhou, A. Characterization of PPIB Interaction in the P3H1 Ternary Complex and Implications for Its Pathological Mutations. *Cell. Mol. Life Sci.* **2019**, *76* (19), 3899–3914. https://doi.org/10.1007/s00018-019-03102-8.

(164) Liaci, A. M.; Steigenberger, B.; Souza, P. C. T. de; Tamara, S.; Gröllers-Mulderij, M.; Ogrissek, P.; Marrink, S. J.; Scheltema, R. A.; Förster, F. Structure of the Human Signal Peptidase Complex Reveals the Determinants for Signal Peptide Cleavage. *Mol. Cell* **2021**, *81* (19), 3934-3948.e11. https://doi.org/10.1016/j.molcel.2021.07.031.

(165) Aydin, S. Multi-Functional Peptide Hormone NUCB2/Nesfatin-1. *Endocrine* **2013**, *44* (2), 312–325. https://doi.org/10.1007/s12020-013-9923-0.

(166) Zhou, Q.; Liu, Y.; Feng, R.; Zhang, W. NUCB2: Roles in Physiology and Pathology. *J. Physiol. Biochem.* **2022**, *78* (3), 603–617. https://doi.org/10.1007/s13105-022-00895-4.

(167) Zheng, L.; Liu, Z.; Wang, Y.; Yang, F.; Wang, J.; Huang, W.; Qin, J.; Tian, M.; Cai, X.; Liu, X.; Mo, X.; Gao, N.; Jia, D. Cryo-EM Structures of Human GMPPA–GMPPB Complex Reveal How Cells Maintain GDP-Mannose Homeostasis. *Nat. Struct. Mol. Biol.* **2021**, *28* (5), 1–12. https://doi.org/10.1038/s41594-021-00591-9.

(168) Shergalis, A. G.; Hu, S.; Bankhead, A.; Neamati, N. Role of the ERO1-PDI Interaction in Oxidative Protein Folding and Disease. *Pharmacol. Ther.* **2020**, *210*, 107525. https://doi.org/10.1016/j.pharmthera.2020.107525.

(169) Boname, J. M.; Bloor, S.; Wandel, M. P.; Nathan, J. A.; Antrobus, R.; Dingwell, K. S.; Thurston, T. L.; Smith, D. L.; Smith, J. C.; Randow, F.; Lehner, P. J. Cleavage by Signal Peptide Peptidase Is Required for the Degradation of Selected Tail-Anchored Proteins. *J. Cell Biol.* **2014**, *205* (6), 847–862. https://doi.org/10.1083/jcb.201312009.

(170) Huang, M.; Chen, Y.; Han, D.; Lei, Z.; Chu, X. Role of the Zinc Finger and SCAN Domain-Containing Transcription Factors in Cancer. *Am. J. Cancer Res.* **2019**, *9* (5), 816–836.

(171) Yamazaki, S.; Fujii, T.; Chiba, S.; Shin, H.-W.; Nakayama, K.; Katoh, Y. TXNDC15, an ER-Localized Thioredoxin-like Transmembrane Protein, Contributes to Ciliary Transition Zone Integrity. *J. Cell Sci.* **2024**, *137* (24), jcs262123. https://doi.org/10.1242/jcs.262123.

(172) Lee, S.-H.; Park, S.-W.; Lee, J.-A.; Jang, D.-J. Identification of C4orf32 as a Novel Type I Endoplasmic Reticulum Resident Membrane Protein. *J. Life Sci.* **2019**, *29* (9), 949–954. https://doi.org/10.5352/JLS.2019.29.9.949.

(173) Altschul, S. F.; Gish, W.; Miller, W.; Myers, E. W.; Lipman, D. J. Basic Local Alignment Search Tool. *J. Mol. Biol.* **1990**, *215* (3), 403–410. https://doi.org/10.1016/S0022-2836(05)80360-2.

(174) Camacho, C.; Boratyn, G. M.; Joukov, V.; Vera Alvarez, R.; Madden, T. L. ElasticBLAST: Accelerating Sequence Search via Cloud Computing. *BMC Bioinformatics* **2023**, *24* (1), 117. https://doi.org/10.1186/s12859-023-05245-9.
